# Non-Interventional, Retrospective, Multicenter Study on the Renal Safety of Liposomal Amphotericin B in Critically Ill Patients with Invasive Pulmonary Aspergillosis

**DOI:** 10.3390/jof12070458

**Published:** 2026-06-23

**Authors:** Borja Suberviola, Jose Peral Gutierrez de Ceballos, Maria Jose Asensio Martin, Cruz Soriano Cuesta, Rafael Zaragoza, Lorena Forcelledo, Iratxe Seijas, Miguel Santibanez

**Affiliations:** 1 Intensive Care Department, University Hospital Marques de Valdecilla, Valdecilla Research Institute (IDIVAL), 39008 Santander, Spain; 2 Intensive Care Department, University Hospital Gregorio Marañon, 28007 Madrid, Spain; joseperalguti@hotmail.com; 3 Intensive Care Department, University Hospital La Paz, IdiPAZ, 28046 Madrid, Spain; 4 Intensive Care Department, University Hospital Ramon y Cajal, 28034 Madrid, Spain; 5 Intensive Care Department, University Hospital Dr. Peset, 46017 Valencia, Spain; 6 Intensive Care Department, University Hospital Central de Asturias, 33011 Oviedo, Spain; 7 Intensive Care Department, University Hospital Cruces, 48903 Barakaldo, Spain; 8 Global Health Research Group, Faculty of Nursing, Universidad de Cantabria-Valdecilla Research Institute (IDIVAL), Santander 39008, Spain

**Keywords:** liposomal amphotericin B, acute kidney injury, invasive pulmonary aspergillosis, critically ill patients, nephrotoxicity

## Abstract

**Purpose:** This study aims to determine the incidence of kidney injury associated with liposomal amphotericin B (L-AmB) treatment, based on RIFLE criteria, in patients admitted to the intensive care unit (ICU) with invasive pulmonary aspergillosis (IPA). **Materials and Methods:** A retrospective, multicenter observational study including patients treated with L-AmB for IPA while admitted to the ICU between 1 January 2015, and 31 December 2022. **Results:** A total of 65 patients were included. The prevalence of renal failure was 35.39%. Renal failure was mostly mild and reversible. The need for major surgery (OR 6.71; *p* = 0.121) and concomitant use of other nephrotoxic treatments (OR 2.5; *p* = 0.194) emerged as potential risk factors for the development of renal failure; however, neither association reached statistical significance. Overall mortality was 66.2%, significantly higher in the group with renal failure (82.6% vs. 57.1%; *p* = 0.03). Factors associated with mortality included concomitant use of other nephrotoxic agents (OR 4.51; *p* = 0.024) and development of renal failure (OR 3.66; *p* = 0.068). Duration of L-AmB treatment was not associated with mortality. Regarding creatinine recovery, all patients who developed renal failure but survived showed creatinine levels below 1.5 mg/dL after completion of treatment. **Conclusions:** Renal impairment was common in this high-risk population of critically ill patients, with renal function impairment in one-third of exposed patients, although most cases were mild. In this population, concomitant administration of other nephrotoxic drugs was associated with both renal failure and mortality. Treatment duration with L-AmB was not linked to mortality, and creatinine levels normalized after therapy completion.

## 1. Introduction

Invasive pulmonary aspergillosis (IPA) is a potentially life-threatening disease that classically affects severely immunocompromised individuals, particularly those with prolonged neutropenia. However, it is now recognized that critically ill non-neutropenic patients represent a high-risk population, with a reported IPA prevalence of 12.4%, mainly due to advances in diagnostic procedures [1].

Amphotericin B is a potent polyene antifungal widely used to treat invasive fungal infections. Recent evidence suggests that amphotericin B exerts its antifungal activity not only through pore formation but also by extracting ergosterol from fungal membranes via extramembranous aggregates. Importantly, cholesterol extraction has been implicated in renal toxicity, and these mechanistic insights have guided the development of novel polyene derivatives with reduced cholesterol binding and improved renal safety profiles [2].

The nephrotoxic effects of amphotericin B are well-documented and result from a combination of hemodynamic and cellular mechanisms. One key factor is the vasoconstriction of the afferent arterioles, which leads to a reduction in renal blood flow and subsequent decline in glomerular filtration rate. In addition, amphotericin B exerts direct cytotoxic effects on the renal tubular epithelial cells, particularly within the distal tubules and collecting ducts, compromising tubular integrity and function. These combined mechanisms contribute to the characteristic pattern of acute kidney injury (AKI) observed with amphotericin B administration [3,4].

Liposomal amphotericin B (L-AmB) is an advanced lipid formulation of amphotericin B designed to improve its safety profile without compromising its potent antifungal activity. In this liposomal formulation, amphotericin B is encapsulated within phospholipid vesicles, which reduces its interaction with cholesterol in human cell membranes, thereby decreasing toxicity, particularly in renal tubular cells. Its use in critically ill patients is common due to its broad-spectrum activity, high efficacy, and improved safety profile.

L-AmB reduces the classic nephrotoxicity associated with amphotericin B because liposomal encapsulation limits direct interaction with renal cell membranes. It also provides a slower and more selective drug release, mainly at sites of infection, minimizing renal exposure, and induces less activation of inflammatory pathways, with reduced oxidative damage and inflammatory cell infiltration in renal tissue [5,6,7].

Critically ill patients exhibit physiological alterations that significantly affect the pharmacokinetics of most drugs. However, L-AmB offers certain advantages: an increased volume of distribution with stable plasma concentrations; a prolonged half-life (up to 150 h), allowing sustained exposure even in low-perfusion states; and no need for dose adjustment in hepatic or renal dysfunction, with preserved bioavailability despite hypoalbuminemia or the hypoperfusion typically observed in septic shock [8,9,10].

Literature reports indicate that the incidence of nephrotoxicity with L-AmB is below 20%, compared to over 50% with conventional amphotericin B [11]. However, accurately determining the true incidence of renal impairment and establishing a causal relationship with renal dysfunction is challenging. The available studies in critically ill populations are limited, often include heterogeneous high-risk cohorts, and use variable definitions for renal dysfunction, antifungal treatment indications, and dosing regimens [12]. Moreover, concurrent use of other potentially nephrotoxic agents complicates the attribution of renal impairment to L-AmB alone [13,14,15].

Consequently, reported rates of L-AmB-associated renal dysfunction in the literature range widely from 0.2% [16] to 38% [17]. Given this variability and the lack of robust data in critically ill cohorts, we conducted this study to better characterize the incidence, outcomes, and risk factors associated with L-AmBrelated renal injury in critically ill patients with aspergillosis.

## 2. Patients and Methods

### 2.1. Study Design and Patients

This was a post-authorization, non-interventional study including patients treated with L-AmB for IPA according to the approved label and routine clinical practice. Patients who met the inclusion criteria and initiated treatment with L-AmB between 1 January 2015, and 31 December 2022, were enrolled. The study was conducted in seven Spanish university hospitals.

The study was conducted in accordance with the Declaration of Helsinki, and the protocol was approved by the Cantabria Research Ethics Committee (Comité de Ética de la Investigación de Cantabria) under approval code CO-ES-131-6262 on 10 June 2022. Given its retrospective and observational nature, the Ethics Committee did not consider it necessary to obtain informed consent from participants.

### 2.2. Inclusion Criteria

*Were as follows:*

- Adult patients (≥18 years).
- Meeting at least one criterion indicating risk for IPA according to 2024 consensus definitions from ESGCIP, EFISG, ESICM, ECMM, MSGERC, ISAC, and ISHAM (XX).
- Proven, probable, possible, or putative IPA according to EORTC-MSG [18], AspICU [19], IAPA [20], or CAPA [21] classifications, as appropriate.
- Treatment with L-AmB per label in routine clinical practice for ≥3 consecutive days during ICU stay.
- Serum creatinine assessment prior to L-AmB initiation, at least 3 times per week during treatment until ICU discharge or death, and weekly after treatment until hospital discharge.

### 2.3. Exclusion Criteria 

*Included:*

- APACHE II score > 20.
- Baseline serum creatinine > 1.5 mg/dL.
- Serum potassium < 3.5 mEq/L.
- Use of any other broad-spectrum antifungal (oral, IV, or nebulized) for more than 5 days before initiating L-AmB for the current episode.

### 2.4. Definitions

Renal dysfunction assessment was standardized using the RIFLE criteria, (Risk, Injury, Failure, Loss, End-stage kidney disease), and renal failure was defined as the presence of a RIFLE classification of Injury or higher at the end of treatment with L-AmB.

Clinical efficacy was defined as the complete or partial resolution of the initial signs and symptoms of pulmonary aspergillosis, with no new signs or symptoms and no need for additional antifungal therapies to treat the infection.

Treatment failure was defined as the progression, relapse, or recurrence of nosocomial pneumonia; inadequate resolution of baseline signs and symptoms; discontinuation of the study drug due to resistance of the causative fungal pathogen(s) of the respiratory tract infection; or patient death.

The microbiological response was classified as eradication, presumed eradication, persistence, or presumed persistence. Eradication was defined as at least one lower respiratory tract culture showing no evidence of the microorganism, absence of growth of the organism in culture media, or normalization of galactomannan levels when previously abnormal. Presumed eradication was defined as the absence of an available culture or microbiological test in a patient who achieved clinical cure. Persistence or presumed persistence was defined as either documented persistence of the microorganism or the absence of an available culture or microbiological test in patients with clinical treatment failure.

The definition of concomitant nephrotoxic therapy was based on previously published studies [22], and a nephrotoxic drug was defined as any agent known to be associated with renal injury that had been administered concomitantly with liposomal amphotericin B for at least 48 h.

Finally, according to the guidelines of the European Surgical Association (ESA), major surgery was defined as procedures characterized by greater technical complexity, prolonged operative time, the need for intensive or intermediate care, and higher associated rates of morbidity and mortality [23].

## 3. Statistical Methods

Descriptive statistics included relative frequencies, means with standard deviations (SD), or medians with interquartile ranges (IQR, p25–p75) for non-normally distributed variables. Group comparisons were performed using the chi-square test or Fisher’s exact test for categorical variables, and Student’s *t*-test or the Mann–Whitney U test for continuous variables, as appropriate.

To estimate the prevalence of renal failure (primary objective), point estimates were calculated along with 95% confidence intervals (CIs). Crude and adjusted odds ratios (ORs) for the main confounding variables were obtained via multivariate logistic regression models.

To assess the reversibility of serum creatinine levels in relation to treatment, measurements were compared across four time periods (baseline, during treatment, end of treatment, and post-treatment), stratified by renal failure status. Group comparisons were performed using the independent Student’s *t*-test for means and the Mann–Whitney U test for medians. Normality was evaluated using both the Kolmogorov–Smirnov and Shapiro–Wilk tests; given inconsistent results, both parametric and non-parametric methods were applied, including the Wilcoxon signed-rank test for related samples.

All statistical tests were two-sided with an alpha error of 5%. Data analysis was conducted using SPSS v22.0 (IBM SPSS, Inc., Armonk, NY, USA) and Epidat v3.1 (Consellería de Sanidade, Xunta de Galicia; Pan American Health Organization [PAHO-WHO]; Universidad CES, Colombia).

## 4. Results

### 4.1. Sample Characteristics

The study included data from 65 patients with a mean age of 68.5 years, 75.4% of whom were male. Patients presented with diverse comorbidities, with the most common being COVID-19 infection (58.5%) and hematologic malignancies (29.2%), consistent with the study period. One-third of the patients presented with septic shock according to the Sepsis-3 definitions at the start of treatment; 20% were receiving corticosteroids and up to 9% were receiving biologic therapies. Baseline severity scores at initiation of L-AmB treatment were APACHE II: 14 ± 4.3 and SOFA: 6.9 ± 3.5. These scores were slightly higher at the end of treatment: APACHE II: 17.9 ± 8.3 and SOFA: 7.9 ± 4.1. The mean L-AmB dose was 3.8 mg/kg/day, with a mean treatment duration of 12.3 days.

Table 1 provides a descriptive overview of patient characteristics overall as well as stratified by the presence or absence of renal function parameter elevations during L-AmB treatment. Two groups were identified based on variations in serum creatinine levels during treatment. No significant differences were found in sex distribution or in the presence of comorbidities such as diabetes mellitus, chronic kidney disease, COPD, cirrhosis or hematologic malignancies. The SOFA score at the end of treatment was significantly higher in patients with elevated serum creatinine above the study threshold (9.9) compared to patients with normal levels (6.9) (*p* = 0.004).

### 4.2. Clinical Response

Antifungal treatment failure was observed in 29 of 65 patients (44.6%), a rate similar to that of microbiological persistence (41%). The highest diagnostic yield was obtained from bronchoalveolar lavage (BAL), with 69.2% positivity, and the most frequently identified species was *Aspergillus fumigatus* (57%). Overall mortality was 66.2% (43/65); however, in half of the cases, the attending physicians did not attribute death directly to IPA. Complete clinical efficacy criteria were met in 38.1% of patients with preserved renal function, compared to 21.7% in the group with renal impairment (*p* = 0.14).

### 4.3. Development of Renal Failure

During the observation period, the prevalence of renal failure was 35.4% (23/65); 95% CI (22.99–47.78). According to the RIFLE classification, most cases were in Stage 1 (56.6%, 13/23). Thirty-three patients received additional concomitant nephrotoxic therapies (see Table 2). In this subgroup, renal failure prevalence was 45.5% (15/33); 95% CI (26.95–63.96). Among those receiving L-AmB as the sole nephrotoxic agent (*n* = 32), renal failure prevalence was 25.0% (8/32); 95% CI (8.44–41.57).

Table 3 shows crude and adjusted associations for major clinical variables. Among the studied variables, major surgery showed the strongest association with renal failure: patients undergoing major surgery had a 6.71-fold increased risk (*p* = 0.121), independently of age, sex, baseline SOFA and APACHE II scores, concomitant nephrotoxic therapy, or treatment duration with L-AmB. The second-most-associated factor was the use of concomitant nephrotoxins: adjusted OR = 2.5 (*p* = 0.194). For each additional day of L-AmB treatment, renal failure risk increased by a factor of 1.04, and by 1.31 per week of treatment (*p* = 0.248), irrespective of major surgery, age, sex, baseline severity scores, or concomitant nephrotoxic use.

### 4.4. Mortality Analysis

Overall mortality was 66.2% (43/65); 95% CI (53.88–78.43). The deceased patients had higher scores on the severity scales, more associated comorbidities (particularly liver cirrhosis and hematological diseases), and more frequently experienced associated renal failure. In this regard, in the group of patients who received additional nephrotoxic treatments, mortality reached 78.8% (26/33); 95% CI (63.33–94.25), compared to 53.1% (17/32); 95% CI (34.27–71.98) in the group treated with L-AmB alone.

Table 4 presents a comparative analysis based on vital status, and Table 5 displays crude and adjusted associations between key clinical variables and mortality.

Among the studied factors (see Table 4), concomitant nephrotoxic therapy was most strongly associated with mortality: adjusted OR = 4.51, *p* = 0.024, independently of renal failure, L-AmB treatment duration, major surgery, age, sex, or baseline severity scores. Renal failure also showed an independent association with mortality: adjusted OR = 3.66, *p* = 0.068. Though not statistically significant at the 5% level, this trend suggests a potential independent association. In contrast, L-AmB treatment duration was not associated with mortality: adjusted OR per day = 0.97, per week = 0.82 (*p* = 0.447), independent of major surgery, demographic factors, severity scores, or nephrotoxic co-treatments.

Mortality was significantly higher in the group of patients with renal impairment (82.6%) compared to those without renal function alteration during treatment (57.1%) (*p* = 0.038).

### 4.5. Renal Function Recovery

**Figure 1:**
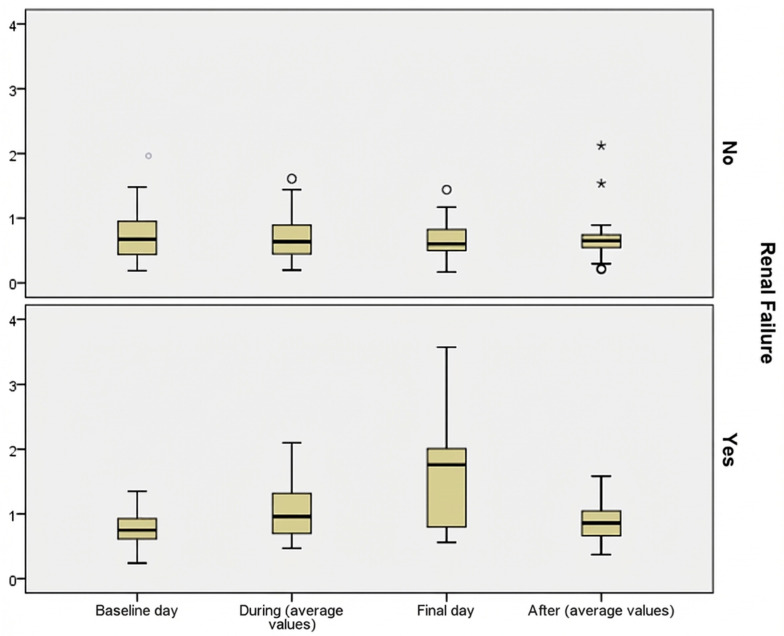
Serum creatinine levels at four time points (baseline, during treatment, end of treatment, and post-treatment) stratified by the presence or absence of AKI. Circles denote mild outliers, defined as observations lying more than 1.5 times the interquartile range (IQR) beyond the quartiles, whereas asterisks denote extreme outliers, lying more than 3 times the IQR beyond the quartiles.

Figure 1 illustrates serum creatinine levels at four time points (baseline, during treatment, end of treatment, and post-treatment), stratified by the presence or absence of AKI. Among patients without renal injury, median creatinine values remained stable. In those with AKI, the highest median was observed on the last day of treatment (1.76 mg/dL), followed by the average during treatment. Median post-treatment and during-treatment creatinine levels were similar (0.96 and 0.86 mg/dL, respectively). As expected, there were no significant differences in baseline or post-treatment creatinine between groups (*p* = 0.32 and 0.082, respectively), while significant differences were noted during treatment and at treatment completion (*p* = 0.001).

As expected, patients with AKI showed significantly different creatinine levels during treatment compared to those without, but there were no significant differences in creatinine levels after completion of treatment (mean values during the hospital stay without amphotericin). However, significant differences were found during treatment and on the day of treatment completion.

## 5. Discussion

This retrospective, multicenter study provides additional evidence on the renal safety of L-AmB in critically ill patients with IPA. The incidence of AKI observed was 35.4%, with most cases classified as mild according to the RIFLE criteria. These findings are consistent with prior studies reporting nephrotoxicity rates around 20% in the general population treated with L-AmB [24], although rates tend to be higher among critically ill patients due to concomitant risk factors [14,25,26], as is the case with the population included in our study.

Several studies have evaluated the nephrotoxic risks associated with L-AmB treatment [27,28,29,30]. In a retrospective multicenter Japanese study, Takazono et al. identified AKI in 37% of patients receiving L-AmB, with most cases corresponding to Stage 1 according to the RIFLE criteria [31]. Similarly, Ueda et al., in a smaller cohort, reported an AKI incidence of 36%, with 80.6% being Stage 1 [32]. In a retrospective study of patients with hematologic malignancies receiving L-AmB, Stanzani et al. found that 38.9% developed some degree of renal injury per the RIFLE scale [27].

Burnett et al. investigated the safety of outpatient L-AmB therapy. While hypokalemia and AKI were common—occurring in 62% and 48% of patients, respectively—only 12% required hospital readmission due to L-AmB-related adverse effects [33].

Hachem et al. reported a lower incidence of nephrotoxicity with L-AmB in patients with hematologic malignancies, both in primary (2.8%) and salvage (5.9%) settings [34]. Wingard et al. observed similar nephrotoxicity rates in the L-AmB subgroup regardless of the dose administered—29.4% for 3 mg/kg/day and 25.9% for 5 mg/kg/day [35].

Several factors have been identified as contributing to AKI development. In their Japanese study, Takazono et al. identified five such factors: prior use of ACE inhibitors or ARBs, carbapenem treatment, concomitant administration of catecholamines (suggestive of shock), immunosuppressants, and L-AmB dosing ≥ 3.5 mg/kg/day [31]. In contrast, data from Personett et al. indicated that L-AmB dosing did not predict renal recovery, as the degree of renal impairment was not associated with the daily dose at the time of AKI onset [36]. A Spanish multicenter study concluded that L-AmB therapy in ICU patients with preexisting renal dysfunction had minimal additional impact on kidney function, as evidenced by serum creatinine values. These findings reinforce the viability of L-AmB as a treatment for IFI in critically ill patients, regardless of their baseline renal function—consistent with hypotheses proposed by Álvarez-Lerma [37].

In Takazono’s study, serum potassium levels below 3.5 mEq/L before initiating L-AmB therapy were associated with severe AKI (Stages 2 and 3) (OR 1.828; 95% CI 1.007–3.319) [31]. Tashiro et al. also found that lower serum potassium levels during hospitalization after L-AmB initiation were associated with AKI [38]. Ueda et al. went further and proposed a “hypokalemia index” as a promising early predictor for AKI [32].

In our study, the concomitant use of other nephrotoxic agents in patients treated with L-AmB was associated with both renal failure and mortality. However, due to the study’s design, it is not possible to determine whether L-AmB itself is more nephrotoxic or more strongly associated with mortality than the concomitant nephrotoxins, as all patients included in the study received L-AmB and there was no comparison group.

What could be determined is the clinical impact of the number of days of L-AmB treatment, which appeared to have limited effect on the development of renal failure, and our findings did not suggest an association with mortality. However, renal failure itself—independent of treatment duration or the use of concomitant nephrotoxins—was associated with increased mortality.

The observed link between concomitant use of other nephrotoxic agents and AKI development aligns with the existing literature [17,39,40,41]. Previous studies have identified aminoglycosides, vancomycin, ACE inhibitors, and diuretics as significant contributors to nephrotoxicity risk in patients receiving L-AmB [37].

Regarding reversibility, our data show that patients who developed AKI and survived experienced recovery of serum creatinine levels to below 1.5 mg/dL after treatment ended. This is encouraging and consistent with previous reports suggesting that L-AmB-induced nephrotoxicity is often partially or fully reversible, especially when risk factors are quickly identified and managed [41,42].

Overall mortality in our cohort was 66.2%, significantly higher in patients who developed AKI (82.6% vs. 57.1%). Although the duration of L-AmB therapy was not directly linked to mortality, the presence of AKI and concomitant use of other nephrotoxic drugs were. These results highlight the importance of closely monitoring renal function and carefully evaluating the need for concurrent nephrotoxic therapies in critically ill patients.

This study has several limitations, including its retrospective design, limited sample size, and the potential for unmeasured confounders. Nevertheless, the use of standardized renal function assessment via RIFLE criteria and the inclusion of multiple centers add robustness to our findings. The small sample size (*n* = 65; 23 with renal failure) likely limited the statistical power, such that some associations of potentially important magnitude (e.g., OR = 3.66) did not reach statistical significance. There was no control group, as all patients received L-AmB. The study population was highly selected, critically ill, and complex, with a very high mortality rate exceeding 60%, which accounts for patient losses during study development. Nonetheless, this approach allows clearer insight into the effects under investigation. Furthermore, few studies to date have specifically addressed L-AmB-induced nephrotoxicity in critical care settings. Due to the retrospective nature of the study, novel biomarkers of renal injury were not included. However, in future prospective investigations, these biomarkers could provide valuable information about which patients are developing true versus pseudo-AKI.

## 6. Conclusions

In conclusion, treatment with L-AmB in critically ill patients with IPA was associated with a moderate incidence of AKI, most cases being mild and reversible. Concomitant nephrotoxic agents showed a possible association with nephrotoxicity, whereas treatment duration did not appear to increase the risk of renal failure. Close monitoring of renal function remains essential, particularly in patients with identifiable risk factors. Further studies are needed to better define the independent contribution of L-AmB to renal toxicity.

## Figures and Tables

**Table 1:** Baseline characteristics of the study population according to renal failure (RIFLE criteria).

Variable	All Patients (*n* = 65)	No Renal Failure (*n* = 42)	Renal Failure (*n* = 23)	*p* Value
Age, years (mean ± SD)	68.5	60.6	64.6	0.044
Male sex, *n* (%)	49 (75.4)	30 (71.4)	19 (82.6)	0.31
Diabetes mellitus, *n* (%)	18 (27.7)	11 (26.2)	7 (30.4)	0.71
Chronic kidney disease, *n* (%)	4 (6.2)	2 (4.8)	2 (8.7)	0.52
Hematologic malignancy, *n* (%)	19 (29.2)	11 (26.2)	8 (34.8)	0.46
COVID-19 infection, *n* (%)	38 (58.5)	25 (59.5)	13 (56.5)	0.81
SOFA score at end of treatment (mean ± SD)	7.9 ± 4.1	6.9 ± 3.6	9.9 ± 4.3	0.004
Concomitant nephrotoxic therapy, *n* (%)	33 (50.8)	18 (42.9)	15 (65.2)	0.08
Mortality, *n* (%)	43 (66.2)	24 (57.1)	19 (82.6)	0.038

**Table 2:** Association between clinical variables and renal failure.

Variable	OR	95% CI	*p* Value	Adjusted OR	95% CI	*p* Value
Concomitant nephrotoxic therapy	2.50	0.87–7.17	0.088	2.17	0.67–6.98	0.194
Major surgery	6.15	0.60–62.92	0.126	6.71	0.61–74.53	0.121
Female sex	0.53	0.15–1.87	0.322	0.70	0.16–3.01	0.628

**Table 3:** Factors associated with mortality.

Variable	Survivors (*n* = 22)	Non-Survivors (*n* = 43)	*p* Value
Hematologic malignancy, *n* (%)	3 (15.8)	16 (84.2)	0.048
Allogeneic HSCT, *n* (%)	0 (0)	7 (100)	0.045
Renal failure, *n* (%)	4 (17.4)	19 (82.6)	0.038

**Table 4:** Association between clinical variables and mortality.

Variable	OR	95% CI	*p* Value	Adjusted OR	95% CI	*p* Value
Renal failure	3.56	1.03–12.30	0.045	3.66	0.91–14.72	0.068
Concomitant nephrotoxic therapy	3.28	1.11–9.71	0.032	4.51	1.22–16.67	0.024

**Table 5:** Serum creatinine values at four time periods according to renal failure.

Time point	No Renal Failure	Renal Failure	All Patients	*p* Value
Baseline creatinine (mean ± SD)	0.71 ± 0.34	0.78 ± 0.29	0.73 ± 0.32	0.387
During treatment (mean ± SD)	0.68 ± 0.32	1.07 ± 0.49	0.81 ± 0.42	0.004
End of treatment (mean ± SD)	0.65 ± 0.31	1.66 ± 0.96	0.96 ± 0.75	0.013
Post-treatment (mean ± SD)	0.70 ± 0.41	0.89 ± 0.37	0.75 ± 0.40	0.269

## Data Availability

The datasets generated and/or analyzed during the current study are available from the corresponding author upon reasonable request. Data are stored in electronic format using Microsoft Excel and SPSS software.

## Abbreviations

AKI	Acute kidney injury
APACHE II	Acute Physiology and Chronic Health Evaluation II
BAL	Bronchoalveolar lavage
CAPA	COVID-19–associated pulmonary aspergillosis
CI	Confidence interval
COPD	Chronic obstructive pulmonary disease
EORTC-MSG	European Organization for Research and Treatment of Cancer/Mycoses Study Group
ICU	Intensive care unit
IAPA	Influenza-associated pulmonary aspergillosis
IPA	Invasive pulmonary aspergillosis
IQR	Interquartile range
L-AmB	Liposomal amphotericin B
OR	Odds ratio
RIFLE	Risk, Injury, Failure, Loss, End-stage kidney disease
SD	Standard deviation
SOFA	Sequential Organ Failure Assessment
